# H9N2 influenza virus acquires intravenous pathogenicity on the introduction of a pair of di-basic amino acid residues at the cleavage site of the hemagglutinin and consecutive passages in chickens

**DOI:** 10.1186/1743-422X-8-64

**Published:** 2011-02-10

**Authors:** Kosuke Soda, Shingo Asakura, Masatoshi Okamatsu, Yoshihiro Sakoda, Hiroshi kida

**Affiliations:** 1 Laboratory of Microbiology, Department of Disease Control, Graduate School of Veterinary Medicine, Hokkaido University, Sapporo, Hokkaido 060-0818, Japan; 2 Research Center for Zoonosis Control, Hokkaido University, Sapporo, Hokkaido 001-0020, Japan; 3 SORST, Japan Science and Technology Agency (JST), 4-1-8 Honcho Kawaguchi, Saitama 331-0012, Japan

## Abstract

**Background:**

Outbreaks of avian influenza (AI) caused by infection with low pathogenic H9N2 viruses have occurred in poultry, resulting in serious economic losses in Asia and the Middle East. It has been difficult to eradicate the H9N2 virus because of its low pathogenicity, frequently causing in apparent infection. It is important for the control of AI to assess whether the H9N2 virus acquires pathogenicity as H5 and H7 viruses. In the present study, we investigated whether a non-pathogenic H9N2 virus, A/chicken/Yokohama/aq-55/2001 (Y55) (H9N2), acquires pathogenicity in chickens when a pair of di-basic amino acid residues is introduced at the cleavage site of its HA molecule.

**Results:**

rgY55sub (H9N2), which had four basic amino acid residues at the HA cleavage site, replicated in MDCK cells in the absence of trypsin after six consecutive passages in the air sacs of chicks, and acquired intravenous pathogenicity to chicken after four additional passages. More than 75% of chickens inoculated intravenously with the passaged virus, rgY55sub-P10 (H9N2), died, indicating that it is pathogenic comparable to that of highly pathogenic avian influenza viruses (HPAIVs) defined by World Organization for Animal Health (OIE). The chickens inoculated with the virus via the intranasal route, however, survived without showing any clinical signs. On the other hand, an avirulent H5N1 strain, A/duck/Hokkaido/Vac-1/2004 (Vac1) (H5N1), acquired intranasal pathogenicity after a pair of di-basic amino acid residues was introduced into the cleavage site of the HA, followed by two passages by air sac inoculation in chicks.

**Conclusion:**

The present results demonstrate that an H9N2 virus has the potential to acquire intravenous pathogenicity in chickens although the morbidity via the nasal route of infection is lower than that of H5N1 HPAIV.

## Background

Each of the known subtypes of the influenza A virus (H1 to H16 and N1 to N9) is circulating in water birds, especially in migratory ducks [1]. A highly pathogenic avian influenza virus (HPAIV) is generated when a non-pathogenic virus brought in by migratory birds from nesting lakes in the north is transmitted to chickens via domestic ducks, geese, quails, turkeys, etc. and acquires pathogenicity for chickens with repeated multiple infections in the chicken population [2-6]. The hemagglutinins (HAs) of HPAIVs differ from those of low pathogenic avian influenza viruses (LPAIVs) with a pair of di-basic amino acid residues at their cleavage site [7]. This structure permits ubiquitous proteases such as furin and PC6, which recognize multiple basic amino acids, to cleave the HA, leading to systemic infection in chickens. By contrast, HAs of LPAIVs are cleaved only by trypsin-like proteases which are expressed in the cells lining the respiratory or intestinal tracts, so that the viruses cause only localized infections, resulting in mild or asymptomatic diseases. It is presently believed that the strains only with H5 or H7 HAs become HPAIVs during extensive infections in chicken populations [8]. The reason why the subtypes of HPAIVs are restricted to H5 and H7 is not known although a model demonstrating that H5 HA is cleaved by furin through molecular docking analyses have been proposed [9,10].

H9N2 avian influenza virus strains have caused outbreaks in poultry, resulting in serious economic losses in Asia and the Middle East [11-19]. The causal strains, however, are avirulent and none of them have multiple basic amino acid residues at the cleavage site of the HA [12,15]. No specific-pathogen-free chickens experimentally infected with H9N2 isolates from diseased chickens showed any clinical symptoms [20]. Co-infection of H9N2 viruses with bacteria such as *Staphylococcus aureus *and *Haemophilus paragallinarum *or with attenuated coronavirus vaccine exacerbated the disease [19,21-23].

Since H9N2 viruses have been isolated not only from domestic birds but also from pigs and humans, the H9 virus has the potential to cause a next pandemic in humans [17,24-27]. It is important for controlling avian influenza and for preparing for pandemic influenza to assess whether the H9N2 virus aquires pathogenicity as H5 and H7 viruses. In the present study, we introduced a pair of di-basic amino acid residues into the cleavage site of the H9 and H5 HAs of non-pathogenic strains. These mutant H9 and H5 viruses were then serially passaged in the air sacs of chicks and their pathogenicity was assessed by inoculation to four-week-old chickens via intravenous and intranasal routes.

## Results

### Generation and characterization of mutant viruses

**Figure 1:**
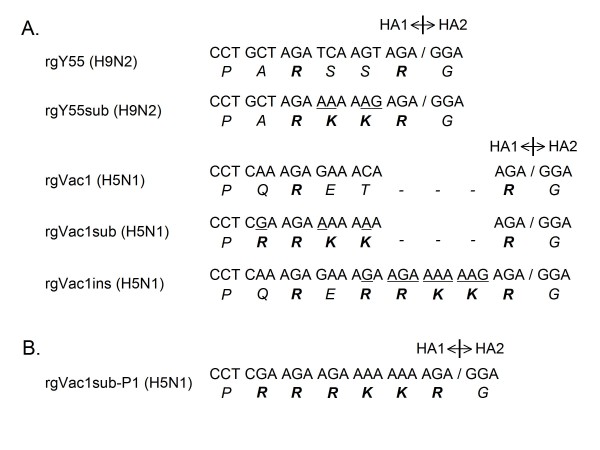
**Nucleotide and amino acid sequences at the HA cleavage site of each virus**. (A) Mutations were introduced into the HA genes of rgY55 (H9N2) and rgVac1 (H5N1) strains to give multibasic amino acids at their cleavage sites by site-directed mutagenesis. Mutation sites were underlined and deduced amino acid sequences were depicted in italics. Basic amino acids were shown in bold. (B) Nucleotide and amino acid sequences at the HA cleavage site of rgVac1sub-P1 (H5N1). rgVac1sub (H5N1) acquired an additional argenine residue represented by the codon AGA after one passage in chickens.

To investigate whether a non-pathogenic H9 influenza virus, A/chicken/Yokohama/aq-55/2001 (Y55) (H9N2) acquires pathogenicity on the introduction of a pair of di-basic amino acid residues at their HA cleavage site, rgY55sub (H9N2) was generated by site-directed-mutagenesis and reverse genetics. Amino acid sequences at the HA cleavage site of the mutant strain are shown in Figure 1A. The RKKR motif was introduced into the H9 HA cleavage site to give a pair of di-basic amino acid residues that is known to be a *sine qua non *for H5 and H7 viruses to become highly pathogenic to chickens. The virus with the insertion of basic amino acid residues at the H9 HA cleavage site was not rescued from plasmid-transfected cells (data not shown). As a positive control, rgVac1ins (H5N1) was generated by inserting the RRKKR motif, rather than RKKR, into the HA of the non-pathogenic virus A/duck/Hokkaido/Vac-1/2004 (Vac1) (H5N1) since recent H5 HPAIV isolates have the motif as insertion mutation. rgVac1sub (H5N1) was also generated to examine whether subtitution mutation with basic amino acid residues at the HA cleavage site contributed to acquisition of pathogenicity for chickens.

rgY55sub (H9N2) and rgVac1ins (H5N1) required trypsin to replicate in MDCK cells, and showed similar levels of growth to their parental viruses (Table 1). Chickens intravenously inoculated with rgY55sub (H9N2) or rgVac1ins (H5N1) did not show any signs of disease. rgVac1sub (H5N1) replicated in MDCK cells without exogenous trypsin, and one of the eight chickens inoculated with the virus showed slight depression at one day post-infection.

**Table 1:** Growth potential in MDCK cells and pathogenicity for chicken of each virus

Viruses	Plaque formation (log PFU/ml)		Pathogenicity (number of dead/sick/total )	
		
	With trypsin	Without trypsin	3-day-old chicks (air sac inoculation)	4-week-old chickens (intravenous inoculation)
rgY55 (H9N2)	8.1	-^a^	NT^b^	0/0/8
rgY55sub (H9N2)	8.0	-	0/0/3	0/0/8
rgY55sub-P5 (H9N2)	7.6	-	0/0/4	0/0/8
rgY55sub-P6 (H9N2)	7.2	6.7	3/3/3	0/1/8
rgY55sub-P7 (H9N2)	7.8	7.5	3/3/3	0/5/8
rgY55sub-P8 (H9N2)	7.6	7.6	3/3/3	2/7/8 (4.0)^c^
rgY55sub-P9 (H9N2)	7.2	6.9	3/3/3	1/8/8 (10.0)
rgY55sub-P10 (H9N2)	6.5	6.1	3/3/3	6/8/8 (1.8)
				
rgVac1 (H5N1)	7.6	-	NT	0/0/8
rgVac1sub (H5N1)	7.6	7.8	0/0/3	0/1/8
rgVac1sub-P1 (H5N1)	6.8	6.8	3/3/3	3/5/8 (5.3)
rgVac1sub-P2 (H5N1)	6.4	6.5	6/6/6	8/8/8 (2.6)
rgVac1ins (H5N1)	7.3	-	0/0/3	0/0/8
rgVac1ins-P1 (H5N1)	7.8	7.1	3/3/3	6/7/8 (6.8)
rgVac1ins-P2 (H5N1)	7.1	7.1	4/4/4	6/8/8 (4.4)

^a ^A plaque was not observed.

^b ^Not tested.

^c ^Mean death days are shown in parentheses.

### Consecutive passages of the viruses in the air sacs of chicks

The H9 mutant virus was serially passaged in the air sacs of chicks to assess whether it acquires pathogenicity as did H5 viruses. The passaged viruses were tested for their growth potential in MDCK cells and pathogenicity for chickens (Table 1). rgY55sub (H9N2) replicated in MDCK cells in the absence of trypsin and killed all of the chicks after six consecutive passages. Two of the eight four-week-old chickens inoculated intravenously with rgY55sub-P8 (H9N2) died within five days. Consequently, over 75% of the chickens intravenously infected with rgY55sub-P10 (H9N2) died by two days post inoculation, and its pathogenicity was comparable to that of the known HPAIVs [28].

H5N1 mutant viruses acquired intravenous pathogenicity by passaging twice in chicks; all of the chickens died after intravenous inoculation with rgVac1sub-P2 (H5N1) or rgVac1ins-P2 (H5N1).

### Amino acid changes of the viruses during consecutive passages in the air sacs of chicks

Nucleotide sequences of the eight segmented genomes of the viruses passaged in the air sacs of chicks were analyzed and compared with those of each parental virus. Leu234 (equivalent to position 226 of H3 HA) in the HA of rgY55sub (H9N2) was substituted with glutamine at the initial passage (Table 2). No other amino acid change was observed up to the fifth passage. Four amino acids in the HA, NA and M2 changed at the sixth passage. One of the asparagine-linked glycosylation sites on the HA was lost by Asn29His mutation. In total, eight amino acid differences were found between rgY55sub (H9N2) and rgY55sub-P10 (H9N2). Five and one amino acid changes were found in the PA, HA, M1 and M2 of rgVac1sub-P2 (H5N1), and the HA of rgVac1ins-P2 (H5N1), respectively (Table 3). It is worth noting that one argenine was inserted at the HA cleavage site of rgVac1sub (H5N1) after one passage in chickens (Figure 1B).

**Table 2:** Amino acid changes during consecutive passages of rgY55sub (H9N2)

Passage number	PB2	HA				NP	NA	M2
					
	271^a^	29	234	357	391	54	195	51
P0	T	N	L	A	N	W	T	I

P1-P5	.^b^	.	Q	.	.	.	.	.
P6	.	H	Q	D	.	.	A	T
P7	.	H	Q	D	N/D^c^	.	A	T
P8	T/A	H	Q	D	N/D	.	A	T
P9	T/A	H	Q	D	N/D	G	A	T
P10	A	H	Q	D	D	G	A	T

^a ^Methionine encoded by the AUG start codon is defined as position 1.

^b ^Periods indicate same amino acids as the parental virus.

^c ^Amino acid quasispecies are observed.

**Table 3:** Amino acid changes during consecutive passages of rgVac1 mutants

Passage number	rgVac1sub (H5N1)									rgVac1ins (H5N1)				
		
	PA		HA		NP		M1		M2		HA		NA	M1
								
	65^a^	672	308	338	213	374	89	101	45	157	298	465	171	130
P0	S	L	H	-	R	M	D	R	R	S	M	D	N	L

P1	.^b^	.	Q	R^c^	.	.	.	.	H	P	.	E	.	.
P2	T	.	Q	R	.	.	.	K	H	P	.	.	.	.
P3	.	F	Q	R	Q	V	N	K	H	P	I	.	N/H^d^	I

^a ^Methionine encoded by the AUG start codon is defined as position 1.

^b ^Periods indicate same amino acids as the parental virus.

^c ^Arginine was inserted at the HA cleavage site.

^d ^Amino acid quasispecies were observed.

### Pathogenicity of the viruses on intranasal infection in chickens

To examine whether the pathogenicity of each virus via the intranasal route of infection correlates with that via intravenous route, three 4-week-old chickens were intranasally inoculated with the viruses of 10^6.5 ^50% egg infectious dose (EID_50_) and observed for clinical signs until day 14 post-infection (Table 4). All chickens inoculated with rgY55sub-P10 (H9N2) or its parental rgY55sub (H9N2) survived without showing any clinical signs, and serum antibodies were detected (1:128-2,048 HI titers), indicating that virus replication occurred.

**Table 4:** Pathogenicity of each virus for chicken via intranasal route

Inoculated viruses	Seroconversion at 14 d.p.i.^a^	Clinical signs	Mortality (dead days)
rgY55 (H9N2)	3/3^b^	0/3	0/3
rgY55sub (H9N2)	3/3	0/3	0/3
rgY55sub-P10 (H9N2)	3/3	0/3	0/3
			
rgVac1 (H5N1)	0/3	0/3	0/3
			
rgVac1sub (H5N1)	1/3	0/3	0/3
rgVac1sub-P2 (H5N1)	0/1	2/3	2/3 (4, 11)
rgVac1sub-P3 (H5N1)	1/1	3/3	2/3 (7, 8)
			
rgVac1ins (H5N1)	1/3	0/3	0/3
rgVac1ins-P2 (H5N1)	1/1	2/3	2/3 (8, 11)
rgVac1ins-P3 (H5N1)	0/1	2/3	2/3 (4, 6)

^a ^Examined for the survived chickens by HI test and ELISA.

^b ^The number of positive animals/total

One of the three chickens inoculated with rgVac1sub (H5N1) or rgVac1ins (H5N1) showed seroconversion after 14 days while no chickens were susceptible to infection with rgVac1 (H5N1). Both of rgVac1sub-P2 (H5N1) and rgVac1ins-P2 (H5N1) were pathogenic, killing two of the three chickens by day 11 post-inoculation.

### Additional passages of the Vac1-based viruses in chickens

One of the three chickens intranasally inoculated with rgVac1sub-P2 (H5N1) or rgVac1ins-P2 (H5N1) did not show any clinical signs (Table 4), indicating that the viruses did not extensively replicate in chickens. rgVac1sub-P3 (H5N1) and rgVac1ins-P3 (H5N1) were prepared from the brain homogenates of the chickens that died on day 11 post-intranasal inoculation with the P2 viruses. Additional amino acid changes were found in P3 viruses (Table 3). To investigate whether the P3 viruses show higher pathogenicity in chicken, the viruses were inoculated via intranasal route. Mortality rate of chickens inoculated with the P3 viruses was equal to that with P2 viruses (Table 4).

### Growth potential of the H9N2 and H5N1 viruses in chickens

To investigate whether tissue tropism of the viruses was involved in their pathogenicity, we determined viral titers in the tissue and blood samples from four-week-old chickens intranasally inoculated with each virus on three days post infection (Table 5). rgY55 (H9N2) and rgVac1 (H5N1) were scarcely recovered from the samples, and the mutant strains before passage in chicks showed broader tissue tropism than the parental viruses. None of the chickens inoculated with rgY55sub-P10 (H9N2) showed any signs of disease, and viruses were recovered from each of the samples except the brain and the blood. One of the three chickens inoculated with rgVac1sub-P2 (H5N1) showed clinical signs such as depression, and the viruses were recovered from virtually all of the organs and blood samples. The remaining two did not show disease signs nor the virus was recovered from any of the tissues tested. Two of the three chickens inoculated with rgVac1ins-P2 (H5N1) showed disease signs, one of them died two days post inoculation, and the virus was recovered from almost all samples. P3 viruses efficiently replicated in each of the tested tissues in chickens as compared with P2 viruses. Throughout the study, the viruses were recovered from the brains of all of the chickens showing clinical signs.

**Table 5:** Virus recovery from the chickens intranasally inoculated with each virus

Inoculated viruses	No. of chickens	Days p.i. (Health status)	Virus recovery (log EID_50_/g)						
			
			Brain	Trachea	Lung	Liver	Kidney	Colon	Blood^c^
rgY55 (H9N2)	3	3 (sacrificed)	-, -, -^b^	-, -, ≦ 1.7	-, -, -	-, -, -	-, -, -	-, -, -	-, -, -
rgY55sub (H9N2)	3	3 (sacrificed)	-, -, -	5.5, 5.7, 6.5	-, 6.7, 2.7	-, 2.7, -	-, -, 2.5	-, -, -	-, -, -
rgY55sub-P10 (H9N2)	3	3 (sacrificed)	-, -, -	-, -, 3.3	-, 3.7, 6.0	-, -, 2.5	-, 4.3, 4.5	-, 3.3, 4.5	-, -, -
									
rgVac1 (H5N1)	3	3 (sacrificed)	-, -, -	-, -, -	-, -, -	-, -, -	-, -, -	-, -, -	-, -, -
rgVac1sub (H5N1)	3	3 (sacrificed)	-, -, 2.7	-, -, -	-, -, 2.5	-, -, -	-, -, -	-, -, ≦ 2.0	-, -, -
rgVac1sub-P2 (H5N1)	2	3 (sacrificed)	-, -	-, -	-, -	-, -	-, -	-, -	-, -
	1^a^	3 (sacrificed)	5.0	3.8	5.0	5.2	7.5	5.2	4.3
rgVac1sub-P3 (H5N1)	2^a^	3 (dead)	5.7, 6.7	5.3, 6.5	4.7, 8.2	3.5, 6.0	7.3, 9.0	5.5, 6.5	NA^d^
	1^a^	3 (sacrificed)	6.3	6.5	6.7	6.5	6.5	6.5	5.8
rgVac1ins (H5N1)	3	3 (sacrificed)	-, ≦ 2.6, -	-, 3.5, -	-,3.3, 3.7	-, -, 3.5	-, 2.7, 3.0	-, 3.0, 2.5	-, ≦ 1.6, 2.8
rgVac1ins-P2 (H5N1)	1^a^	2 (dead)	3.5	3.4	4.7	3.7	4.8	4.7	NA
	1^a^	3 (sacrificed)	3.8	3.7	3.0	≦ 2.0	4.7	≦ 2.0	-
	1	3 (sacrificed)	-	-	-	-	-	-	-
rgVac1ins-P3 (H5N1)	3^a^	3 (sacrificed)	3.4, 4.7, 5.5	4.7, 3.5, 4.2	5.5, 5.2, 6.7	4.3, 4.5, 5.7	4.5, 4.7, 5.2	5.3, 4.5, 5.3	3.0, 2.5, 3.5

^a ^Each chicken showed depression.

^b ^1.5 ≧ (0.5 ≧ for blood samples)

^c ^log EID50/ml

^d ^Not applicable.

## Discussion

Here, we demonstrated that the H9N2 influenza virus acquired intravenous pathogenicity after a pair of di-basic amino acid residues was introduced into the cleavage site of the HA and serially passaged in chicks. Since rgY55sub-P10 (H9N2) killed 75% of chickens inoculated via intravenous route, the pathogenicity was comparable to that of HPAIVs (Table 1). On the other hand, chickens intranasally inoculated with rgY55sub-P10 (H9N2) did not show any clinical signs of disease (Table 4). These results are consistent with those of previous study showing that some H10 influenza viruses did not show intranasal pathogenicity for chicken while their intravenous pathogenicity index was over 1.2 and classified as HPAIV according to the definition by European Union [29]. Amino acid changes during consecutive passages in the air sacs of chicks (Table 2) are considered to be responsible for the acquisition of intravenous pathogenicity, and their effects on the functions of viral proteins should be clarified further. Here we focused on two substitutions at positions 29 and 234 of the H9 HA molecule. It has been reported that residue 226, based on the H3 HA numbering (234 in the present study), relates to receptor specificity and cell tropism [30]. Strain Y55 (H9N2) originally had a leucine at this position, and the change to glutamine after serial passages in the air sacs of chicks indicates that the passaged rgY55sub (H9N2) was further adapted to chicken. One of the asparagine-linked glycosylation sites on the HA of rgY55 (H9N2) lost a carbohydrate attachment with the substitution of Asn29His. The site locates sterically in the vicinity of the HA cleavage site, suggesting that the deletion of the carbohydrate chain affected the susceptibility of the HA to the host protease [31]. This notion is also supported by the present finding that the rgY55sub viruses (H9N2) after six passages in the air sacs of chicks replicated in MDCK cells in the absence of trypsin (Table 1). Ohuchi et al. (1991) reported that the insertion of additional basic amino acids into the H3 HA cleavage site resulted in intracellular proteolytic cleavage. Other groups reported that H3 and H6 HAs tolerated amino acid mutations into their cleavage sites and the viruses with the mutated HAs replicated in MDCK and/or QT6 cells in the absence of trypsin [32,33]. The results in the present study are in agreement with these, namely, cleavage-based activation by an ubiquitous protease is not restricted to the H5 and H7 HAs.

rgVac1sub (H5N1) and rgVac1ins (H5N1) acquired marked intravenous and intranasal pathogenicity after a few passages in chicks (Table 1). It was reported that an avirulent H5 virus isolated from a swan became highly pathogenic in chickens after 24 consecutive passages in the air sacs, followed by five passages in the brains of chickens [3]. The differences in time required for the viruses to become highly pathogenic between these studies depended on the amino acid motif at the HA cleavage site prior to passaging. rgVac1sub (H5N1) acquired an arginine at the HA cleavage site after only one passage in chickens (Figure 1B and Table 3), suggesting that an additional insertion of basic amino acid residues efficiently occurred in the serial basic amino acid residues at the cleavage site. One third of the chickens inoculated intranasally with rgVac1sub-P2 (H5N1) or rgVac1ins-P2 (H5N1) survived 14 days (Table 4). In addition, one of the birds was not susceptible to infection with rgVac1sub-P2 (H5N1), indicating that viral replication may depend on the presense of P3-like viruses in the inoculum.

The intranasal pathogenicity of the mutants of H9N2 virus was different from those of H5N1 mutants while these viruses replicated in MDCK cells in the absence of trypsin and killed chickens when inoculated via intravenous route (Tables 1 and 4). The viruses were recovered from the brain and the blood of some chickens infected with rgVac1 mutants (H5N1), and morbidity was closely associated with viral titers in the brain (Table 5). No viruses were recovered from the brain and the blood of chickens infected with rgY55 mutants (H9N2), indicating the reason why rgY55sub-P10 (H9N2) did not show intranasal pathogenicity. All the viruses passaged in the air sacs of chicks killed chicken embryos by 48 hours post allantoic inoculation (data not shown). rgVac1sub-P3 (H5N1) and rgVac1ins-P3 (H5N1) were more pathogenic to chicken embryos than rgY55sub-P10 (H9N2); the allantoic fluids obtained from the embryonated eggs inoculated with the H5N1 viruses passaged in the air sacs were turbid. It was reported that infection of a highly pathogenic virus was strictly confined to endothelial cells in chicken embryos or chickens [34,35]. Therefore, it is suggested that the difference of endotheliotropism between the H9N2 and H5N1 viruses passaged in the air sacs affected their intranasal pathogenicity. rgY55sub-P10 (H9N2) was not recovered from the brain and the blood of chickens although it caused systemic infection (Table 5), indicating that high levels of viremia followed by replication in the vascular endothelial cells was prerequisite for the virus to cross the blood-brain barrier and consequently replicated in the brain. This hypothesis is supported by the result that rgY55sub-P10 (H9N2) showed intravenous pathogenicity in chickens; direct injection of the virus to the blood vessels readily caused viremia, leading to invasion of the virus to the brain. (Table 1).

H9N2 viruses which have the PARSKR or PARSRR motifs at their HA cleavage site have been isolated from turkeys, ostriches, and chickens in Israel and quails in China [14] although PARSSR motif has been found in most H9N2 isolates, indicating that such substitutions with basic amino acid residues occur in nature. If serine at the c-terminus of the HA1 of the H9 virus was substituted with lysine, the amino acid motif would be consistent with that of rgY55sub (H9N2) which acquired intravenous pathogenicity on consecutive passages in the air sacs of chicks. LPAI caused by H9N2 strains in poultry is now causing serious economic losses [11-19], and its eradication is still difficult because of its low pathogenicity, frequently causing inapparent infections. The present study demonstrated that H9N2 viruses circulating in chicken flocks can acquire intravenous pathogenicity. It is predicted that co-infections of rgY55sub-P10 (H9N2) with bacteria exacerbate not only intravenous pathogenicity but intranasal pathogenicity in chickens as shown in a previous study [21]. Therefore, continuous monitoring in poultry is important to prevent the emergence of pathogenic H9 viruses.

## Materials and methods

### Viruses

A/chicken/Yokohama/aq-55/2001 (Y55) (H9N2) isolated from chicken meat imported from China upon quarantine was kindly provided by Dr. M. Eto, Animal Quarantine Service (Yokohama, Kanagawa, Japan) [36]. A/duck/Hokkaido/Vac-1/2004 (Vac1) (H5N1) was generated by the standard genetic reassortment procedure from non-pathogenic viruses, A/duck/Mongolia/54/2001 (H5N2) and A/duck/Mongolia/47/2001 (H7N1) [37-39]. Viruses were propagated in ten-day-old embryonated chicken eggs for 48 hours at 35°C.

The complete nucleotide sequences of Y55 (H9N2) and Vac1 (H5N1) have been registered in GenBank/EMBL/DDBJ (Accession numbers: AB256671-AB256678 [36] and AB259709-AB259716 [37], respectively).

### Reverse genetics

Viral RNA was extracted from the allantoic fluid of embryonated chicken eggs infected with the Y55 and Vac1 strains using a commercial kit (TRIzol LS Reagent, Sigma-Aldrich, St. Louis, MO, U.S.A) and reverse-transcribed with the Uni12 primer [40] and M-MLV Reverse Transcriptase (Invitrogen, Carlsbad, CA, U.S.A). PCR-based amplification of the full genomes of the eight gene segments was performed with universal primer sets [41]. The PCR products were cloned into the vector pCR2.1-TOPO (Invitrogen) or pGEM-T Easy Vector (Promega, Mannheim, Germany). After confirmatory sequencing, T-vector clones were digested with *BsmBI *and inserted into the vector pHW2000 [42]. MDCK cells and 293T cells were maintained in Minimum Essential Medium (MEM, Nissui Pharmaceutical, Tokyo, Japan) containing 10% calf serum and D-MEM (Invitrogen) containing 10% FBS, respectively. Before transfection, confluent 293T and MDCK cells in 75 cm^2 ^flasks were trypsinized, and 10% of each cell line was mixed in 12 ml of Opti-MEM I (Invitrogen); 2 ml of the suspension was seeded into each well of six-well tissue culture plates (Nunc Inc., Naperville, IL). The cocultured 293T and MDCK cells were used for the transfection. TransIT-293 (Panvera, Madison, WI) was used to transfect cells according to the manufacturer's directions. Briefly, two microliters of TransIT-293 per microgram of DNA was mixed, incubated at room temperature for 45 minutes, and added to the cells. The transfection mixture was replaced with Opti-MEM I after six hours of incubation at 37°C. Thirty hours later, Opti-MEM I containing one microgram per microliter of trypsin was added. At 48 to 72 hours post-transfection, the culture supernatant was collected and propagated in ten-day-old embryonated chicken eggs.

### Site-directed-mutagenesis

To generate H9 and H5 mutant viruses with basic amino acid residue substitutions (sub) or insertions (ins) at the HA cleavage site, mutations were introduced into the HA genes of the Y55 and Vac1 strains using a QuikChange II site-directed mutagenesis kit (Stratagene, Heidelberg, Germany) according to the manufacturer's instructions. The mutant viruses, rgY55sub (H9N2), rgVac1sub (H5N1), and rgVac1ins (H5N1), were rescued by reverse genetics as described above, and the entire genomes of the eight gene segments were sequenced to confirm the existence of the introduced mutations and the absence of undesired mutations.

### Plaque assay

Ten-fold dilutions of viruses were inoculated onto confluent monolayers of MDCK cells and incubated at 35°C for one hour. Unbound viruses were removed by washing the cells with MEM. Cells were then overlaid with MEM containing 0.7% Bacto-agar (Difco, Sparks, MD) in the presence or absence of trypsin (5 μg/ml). After 48 hours of incubation at 35°C, cells were stained with 0.005% neutral red.

### Consecutive passage in the air sacs of chicks

The caudal thoracic air sacs of three 3-day-old chicks were inoculated with 200 μl of each of the mutant Y55 and Vac1 viruses. The chicks were sacrificed, and their lungs and brains were collected at three days post-inoculation. Serial passages in the air sacs of three to six 3-day-old chicks were performed with 200 μl of a pooled 10% tissue suspension of infected organs. Brain samples were used as the inoculum when both samples (lungs and brains) tested positive for the virus. Isolates were identified by their parental strain's name, mutation (substitution or insertion), and number of passages. For example, the designation rgY55sub-P10 (H9N2) indicates that the amino acids at the HA cleavage site of the Y55 virus were substituted with basic amino acids as shown in Figure 1A, then passaged ten times in the air sacs. Passaged viruses were propagated in the allantoic cavities of ten-day-old embryonated chicken eggs for 48 hours at 35°C. The allantoic fluid was harvested and stored at -80°C.

### Experimental infection of chickens with mutant virus strains

Four-week-old Boris Brown chickens were used to test the pathogenicity of the passaged viruses. Eight chickens were intravenously inoculated with 200 μl of each virus (1:10 diluted allantoic fluid), and examined for clinical signs at intervals of 24 hours over a period of ten days. Similarly, three chickens were infected intranasally with 100 μl of allantoic fluid containing each virus at a EID_50 _of 10^6.5 ^and observed for 14 days. Specific antibodies against homologous viruses after 14 days of infection were detected in serum using the hemagglutination inhibition (HI) test and/or enzyme-linked immunosorbent assay (ELISA) as described previously [43]. To study viral replication, each virus was inoculated into three chickens at an EID_50 _of 10^6.5^. The birds were euthenized three days post-challenge, and tissue and blood were collected aseptically. To make a 10% suspension with MEM, the tissue samples were homogenized using a Muti-Beads Shocker (Yasui Kikai, Osaka, Japan). These suspensions were serially diluted ten-fold with PBS and inoculated into ten-day-old embryonated eggs and incubated at 35°C for 48 hours. Viral titers were calculated by the method of Reed and Muench [44] and expressed as EID_50_ per gram and milliliter of tissue and blood, respectively.

All experiments were carried out in self-contained isolator units (Tokiwa Kagaku, Tokyo, Japan) at a BSL3 biosafety facility at the Graduate School of Veterinary Medicine, Hokkaido University, Japan. The experiments were performed according to the guidelines of the institutional animal care and use committee of the Graduate School of Veterinary Medicine.

## Competing interests

The authors declare that they have no competing interests.

## Authors' contributions

HK is the leader of the study group. KS carried out the experiments and wrote the manuscript. SA helped in passaging study. KS, MO, YS, and HK designed the experiments and analyzed the data. All authors read and approved the final manuscript.
